# Supplementary material for: Self-evolving vision transformer for chest X-ray diagnosis through knowledge distillation
Source: Nat Commun. 2022 Jul 4;13:3848. doi: 10.1038/s41467-022-31514-x (PMC9252561; doi:10.1038/s41467-022-31514-x)
Supplement: Supplementary file 1 — Supplementary Information [file 41467_2022_31514_MOESM1_ESM.pdf]

Supplementary Information

Self-evolving vision transformer for chest X-ray diagnosis through knowledge distillation

Sangjoon Park, Gwanghyun Kim, Yujin Oh, Joon Beom Seo, Sang Min Lee, Jin Hwan Kim, Sungjun Moon, Jae-Kwang Lim, Chang Min Park, and Jong Chul Ye

Terminology

Self-supervised learning. Unsupervised learning is a learning approach that learns underlying patterns of data without any pre-existing label. Self-supervised learning is a type of unsupervised learning approach, in which the unlabeled data itself provides the supervisory signal which enables the model to learn underlying patterns of data. Usually, it is formulated from a large data corpus by training the model to learn several pretext tasks, which usually includes matching the differently augmented versions of the same image[1], predicting mask area[2], matching the original image from small cropped lesion[3] and so on. Through the pretext learning, the model can learn to discriminate between similar and dissimilar data, and thereby cluster them without any supervision from label (Supplementary Fig. S1c). After the self-supervised learning, the model can easily adapt to the downstream tasks with a small number of labeled data, as it has already learned task-agnostic visual representation from the pretext tasks.

Self-training. Semi-supervised learning, as the name suggests, is a learning paradigm located somewhere between supervised and unsupervised learning. It is generally used when a small amount of labeled data and a large amount of unlabeled data exist. In semi-supervised learning, a large number of unlabeled data is utilized by referring to the small amount of labeled data. For example, a large number of unlabeled data points can be used to provide a more accurate decision boundary for all data for the classification task (Supplementary Fig. S1d). Self-training is one of the representative semi-supervised learning methods. In this method, a learner (teacher) obtained with supervised learning via small labeled data keeps on labeling large unlabeled data, called pseudo-label. The pseudo-labels, in turn, constitutes an enlarged data corpus, so that one can use the data for retraining the model (student)[4]. In earlier works, it has been shown to be a promising approach to leverage unlabeled data to improve model performances[5–7].

Knowledge distillation. Knowledge distillation is a learning paradigm of transferring the knowledge from the teacher model to the student model, as mentioned above in the explanation of self-training. It was originally developed for model compression, where the aim is to efficiently build the simple student by distilling the knowledge of the complex teacher, often required for practical implementation of AI model in devices with limited computational resources[8]. However, since this

configuration can be utilized in the framework with a siamese design where one model learns from the prediction of the other model instead of labels, some lines of semi- and self-supervised learning works utilized knowledge distillation as mentioned above in the application for self-training. Of note, several recent studies have suggested the possibility that the model can obtain a performance similar to or better than the fully supervised model through semi- or self-supervised learning methods based on the knowledge distillation framework[3,6].

Details of DISTL algorithm

Pre-training for task-relevant findings. As transfer learning from the relevant tasks can significantly improve the performances, we first trained the model to classify the common CXR findings using a large corpus of the CheXpert dataset[9], containing over 220,000 CXRs and corresponding labels. Among 10 common CXR findings, lung opacity, consolidation, edema, pneumonia, and pleural effusion were selected as the task-relevant classes based on the clinician's opinion. With the pre-training for the task-relevant CXR features on massive CXR data, the model can have excellent generalization capability in the external validation. In addition, this pre-training step enables the model to have robust feature extracting capacity with the prior knowledge of CXR. Without the pre-training, the model performances significantly decreased, especially for early time T (Supplementary Fig. S3).

Self-evolving training scheme. Details of our training scheme are illustrated in Fig. 2. First, with the small labeled data $D_l = \{(x_1, y_1), (x_2, y_2), ...(x_n, y_n)\}$, the initial model is built with supervised learning. Then, we used this model as the initial teacher, and let the student learn from the teacher using the proposed DISTL method. In addition, to prevent the student from performance deterioration caused by the wrong estimations, the supervised correction with initial small labeled data is done per N steps. The updated models at the end of the DISTL are utilized as the starting point of the next-generation model, similar to the previous self-training approach[6]. Specifically, as the amount of available unlabeled data D_u^T increases over time $T = 1, 2, ...T$, the updated teacher and student models $g_{\theta_t}^T$, $g_{\theta_s}^T$ at the end of the DISTL for the current T are used as the starting point of the next teacher and student models $g_{\theta_t}^{T+1}$, $g_{\theta_s}^{T+1}$ for $T+1$.

Loss functions for self-supervised and self-training. The overall framework of our method shown in Fig. 1c has a similar configuration to recent self-supervised (Fig. 1b)[3,10] and self-training approaches (Fig. 1a)[6], which also share similarities of the knowledge distillation for teacher-student learning.

Specifically, both the teacher and student models share the same network architecture. The network architecture g parameterized by θ is composed of backbone f (before the final linear classifier) and of two heads h^{cls}, h^{ss} for disease classification and self-supervision, respectively:

$$g_\theta^{cls} = h^{cls} \circ f, \quad g_\theta^{ss} = h^{ss} \circ f \tag{1}$$

Given an input image x, the models in (1) yield two predictions $P_\theta^{cls}(x)$ and $P_\theta^{ss}(x)$ with the dimensions K^{cls} and K^{ss}, respectively, by normalizing the network output with the softmax function with temperature parameter τ^{cls} and τ^{ss}:

$$P_\theta^{cls}(x) = \text{Softmax}\left(g_\theta^{cls}(x)\right), \quad P_\theta^{ss}(x) = \text{Softmax}\left(g_\theta^{ss}(x)\right) \tag{2}$$

where

$$\text{Softmax}\left(g_\theta(x)\right) = \frac{\exp\left(g_\theta(x)/\tau\right)}{\Sigma_{k=1}^{K}\exp\left(g_\theta(x)^{(k)}/\tau\right)} \tag{3}$$

where τ controls the sharpness of the output distribution.

Then, we train a student model parameterized by θ_s to match the prediction of teacher model parameterized θ_t. Specifically, for a given input image x, a set $V = \{x^o, x^g, x_1^l, ...x_L^l\}$ was constructed, containing one original view x^o without augmentation, one global view x^g and L local views x^l of smaller size, where one global crop and multiple local crops are obtained with the multi-crop strategy[11] and random augmentations to construct differently distorted views. We used the set V in two ways. First, the clean original view is passed to the teacher, while the global view with weak augmentation and noises is passed to the student model. Then, the student is trained to mimic the pseudo-label generated by the teacher which minimizes the cross-entropy:

$$\mathcal{L}^{cls} = \min_{\theta_s} \sum_{x \in \{x^o\}} \sum_{x' \in \{x^g\}} - P_{\theta_t}^{cls}(x) \log P_{\theta_s}^{cls}(x') \tag{4}$$

Secondly, the original and global views are passed through the teacher while all views are passed through the student, thereby encouraging global-local correspondence with the following optimization problem:

$$\mathcal{L}^{ss} = \min_{\theta_s} \sum_{x \in \{x^o, x^g\}} \sum_{\substack{x' \in V \\ x' \neq x}} - P^{ss}_{\theta_t}(x) \log P^{ss}_{\theta_s}(x') \tag{5}$$

We found that optimizing this self-supervised term can encourage the model to learn the task-agnostic semantic features of the CXR (Supplementary Fig. S2), which implies that the model better attends to the shape of the CXR as a human reader does.

Combined Eq. (4) and Eq. (5) together, the final optimization problem can be defined using the weighted combination.

$$\mathcal{L} = (1 - \alpha)\mathcal{L}^{cls} + \alpha\mathcal{L}^{ss} \tag{6}$$

where α is a hyperparameter to adjust the weights between classification loss and self-supervising loss.

Unlike the noisy self-training where the teacher model remains unchanged during training, we built a momentum teacher using an exponential moving average (EMA) on the student weights, where λ follows a cosine scheduling:

$$\theta_t = \lambda\theta_t + (1 - \lambda)\theta_s \tag{7}$$

This encourages the update of the student to slowly pervade on the weights of the teacher with momentum, enabling the teacher to improve its performance gradually in accordance with the student as well averting the performance deterioration from the student misguided by wrong predictions.

The two loss functions in (6) deserve further discussion. By minimizing the first term $\mathcal{L}^{cls}$, the noised student is trained to be consistent with the pseudo-label generated by the clean teacher. Adding noise brings an important benefit of forcing invariance in the decision function, as it enforces the student to have prediction consistency across a variously augmented version of a given image. In addition, it can also add robustness to the common corruption and perturbations[6].

The minimization of the second term, $\mathcal{L}^{ss}$, enforces the global-local correspondence so that the model can learn self-supervised features explicitly containing object boundaries and the semantic information[3]. These task-agnostic self-supervised features provide a useful shape-bias like humans that helps avoid the overfitting to the texture and other non-informative characteristics of the image, resulting in the improvement of generalization performance and the stability of the model.

Ablation Study

We performed the ablation studies to clarify the role of each component in the proposed DISTL framework (Supplementary Fig. S3).

Pre-training on task-relevant CXR features. Learning general but task-relevant CXR features from the pre-training on a large data corpus is one of the key components of our method. As shown in Supplementary Fig. S3, when not utilizing the pre-trained weights as an initialization point, the performance was suboptimal, while the gradual performance increased with the proposed framework under the increasing amount of unlabeled data was maintained.

Role of two loss terms. As the loss function of the proposed method consists of two terms, we ablated each term and evaluated the effect. As shown in Supplementary Fig. S3, when not using the self-supervising loss term, the performance was not improved over increasing T and even decreased at later T, devastating the merit of our framework. Similarly, when training the model only with the self-supervising loss term and fine-tuning with the label data at the correction step, the model performance was overall lower than that trained with both terms. Combined, these results suggest that both terms are necessary for the proposed framework to achieve stably improving performance with the increasing unlabeled data.

Correction step with label. The correction step within the proposed framework plays a role in restoring erroneously updated weights from the wrong estimation of the teacher with the initial set of small labeled data. To verify its contribution to the performance, we performed ablation of the

correction step (see Supplementary Fig. S3). While the performance improvement was observed compared to the baseline, the performance improvement over increasing T was lower than that with the correction step.

Supplementary Table. 1. Details of data collection for diagnosis of tuberculosis.

Class	Training and internal validation										
	**Total**	**AMC**	**NIH**	**BIMCV**	**CheXpert**	**India**	**Montgomery**	**Shenzen**	**Belarus**	**PADChest**	**TBX 11K**
Normal	30,092	8,978	7,123	93	9,577	102	92	327	-	-	3,800
Tuberculosis	5,893	497	3,709	-	-	74	46	335	297	135	800
Total	35,985	9,475	10,832	93	9,577	176	138	662	297	135	4,600

AMC, Asan medical center; NIH, National Institutes of Health; BIMCV, Valencian Region Medical ImageBank; PADChest, PAthology Detection in Chest radiograph; TBX 11K, Tuberculosis X-ray 11K.

Supplementary Table. 2. Detailed diagnostic performances of the model trained with the proposed method.

Metrics	Pooled	CNUH	YNU	KNUH
AUC	0.974	0.965	0.985	0.98
(95% CI)	(0.964-0.985)	(0.926-1.000)	(0.968-1.000)	(0.966-0.993)
Sensitivity	92.7	92.9	93.0	95.0
(95% CI)	(89.3-95.3)	(76.5-99.1)	(86.1-97.1)	(91.0-97.6)
Specificity	92.0	90.3	96.0	93.5
(95% CI)	(90.2-93.5)	(86.9-93.0)	(93.1-97.9)	(90.6-95.7)
Accuracy	92.2	90.4	95.3	94.0
(95% CI)	(90.6-93.5)	(87.2-93.0)	(92.7-97.1)	(91.8-95.8)
PPV	0.776	0.400	0.886	0.880
(95% CI)	(0.738-0.809)	(0.327-0.478)	(0.816-0.931)	(0.834-0.914)
NPV	0.977	0.995	0.976	0.974
(95% CI)	(0.966-0.984)	(0.979-0.999)	(0.953-0.988)	(0.953-0.986)

CNUH, Chungnam national university hospital; YNU, Yeungnam University Hospital; KNUH, Kyungpook National University Hospital; AUC, area under the receiver operating characteristics curve; CI, confidence interval.

Supplementary Table. 3. Data partitioning to simulate the experiment of real-world data collection.

Class	Training and internal validation			
	T=initial	T=1	T=2	T=3
	Labeled	Unlabeled		
Normal	3,033	9,013	18,047	27,059
Nodule	-	300	600	900
Effusion	-	260	520	780
ILD	-	290	580	870
Bacterial infection	-	166	332	500
Tuberculosis	565	1,782	3,543	5,328
Total	3,598	11,811	23,622	35,437

T, time; ILD, interstitial lung disease.

Supplementary Table. 4. Data partitioning used to simulate the experiment of label corruption.

Class	Training and internal validation			
	T=initial	T=1	T=2	T=3
	Labeled			
Not corrupted	3,598	10,255	20,510	30,768
Corrupted	-	540	1,080	1,619
Total	3,598	10,795	21,590	32,387

T, time.

Supplementary Table. 5. Detailed diagnostic performances of the model trained with the proposed method in a cohort of bacteriological laboratory-confirmed tuberculosis cases.

Method	AUC (95% CI)	Sensitivity (95% CI)	Specificity (95% CI)	Accuracy (95% CI)	PPV (95% CI)	NPV (95% CI)
Proposed	0.952 (0.940-0.963)	87.7 (84.6-90.3)	89.1 (86.6-91.3)	88.5 (86.6-90.2)	0.856 (0.828-0.880)	0.908 (0.887-0.925)

AUC, area under the receiver operating characteristics curve; PPV, positive predictive value; NPV, negative predictive value; CI, confidence interval.

Supplementary Table. 6. Proportion of negatively diagnosis cases, negative predictive value (NPV) and false positive rate (FPR) in simulation for application.

Method	Negative proportion	NPV	FPR
Proposed	72.50%	0.977	0.08

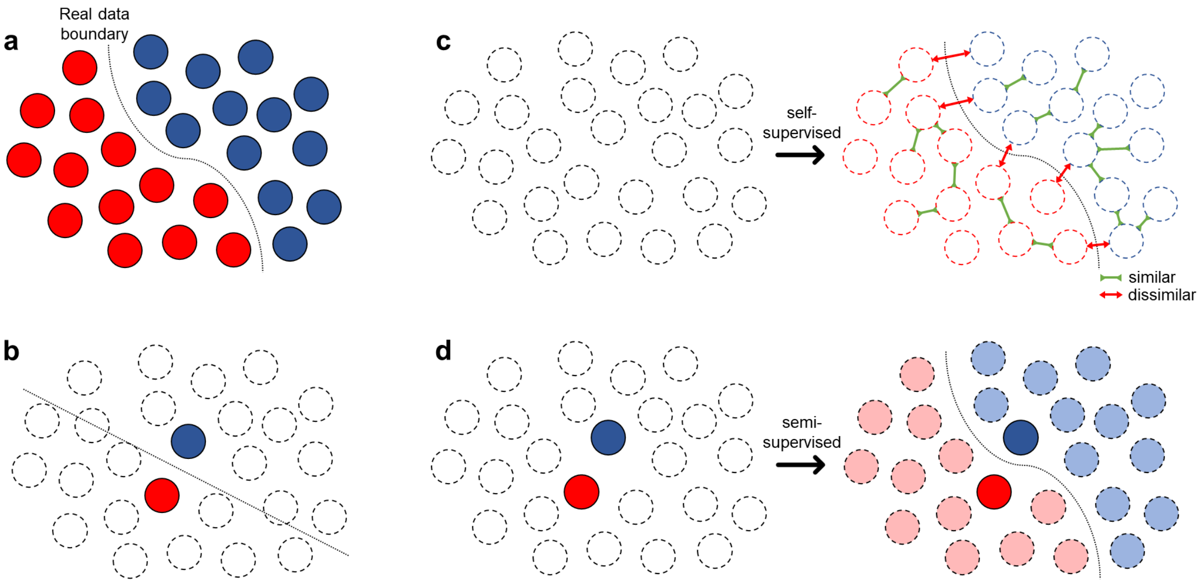

Supplementary Fig. S1. Illustration of the differentc learning methods. The colored circles denote labeled data, while the empty circles denote unlabeled data. (a) Supervised learning with fully labeled data and (b) with the small number of labeled data. (c) Self-supervised with the unlabeled data. Similar data points are clustered without any supervision from label. (d) Semi-supervised learning with the small labeled and large unlabeled data. Unlabeled data are utilized referring to the labeled data points, as painted in faint colors that stand for the pseudo-labels. Given the small number of labels, self- or semi-supervised learning provides more accurate decision boundary than supervised learning.

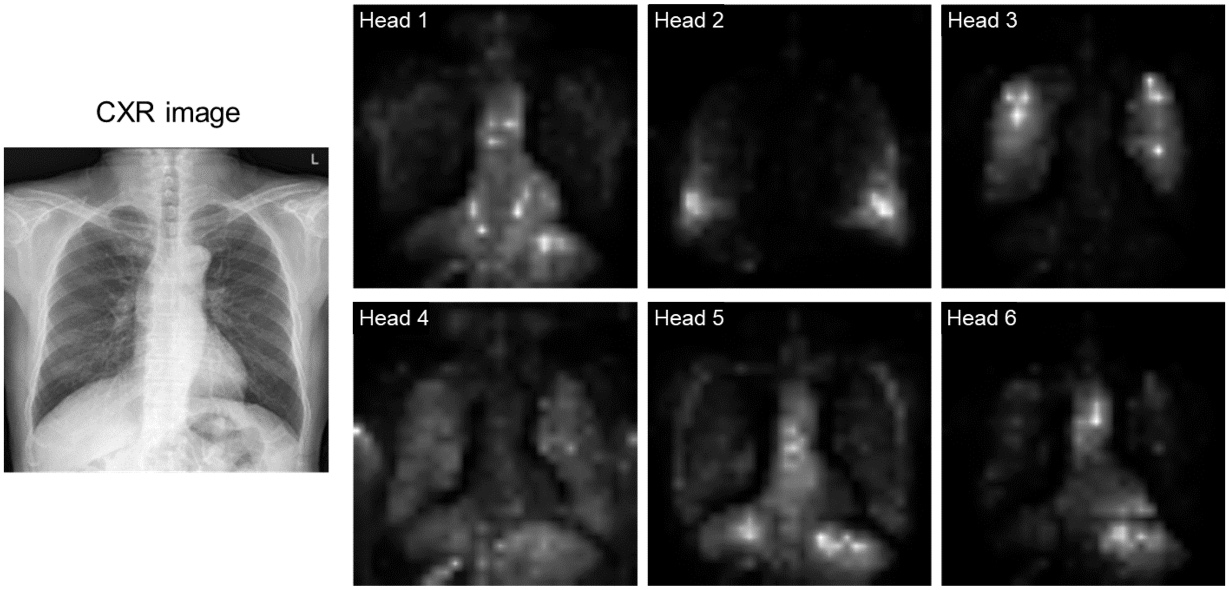

Supplementary Fig. S2. Example of attention obtained with self-supervised learning. The example of attention obtained only with self-supervised learning on a normal chest X-ray (CXR) shows that different attention heads attend to different structures.

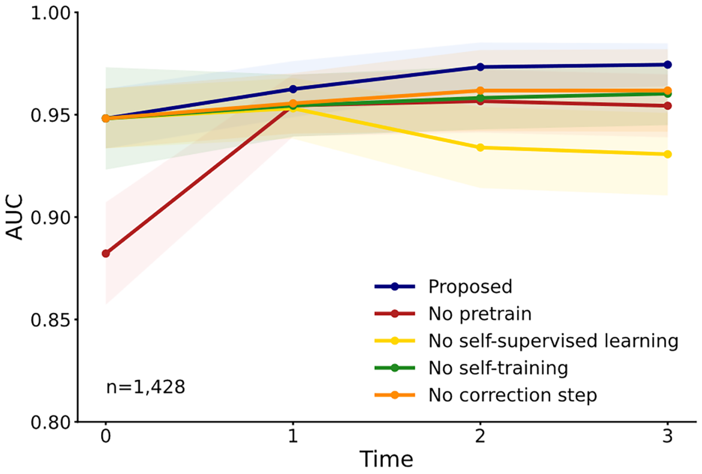

Methods	T = initial	T = 1	T = 2	T = 3
Proposed	0.948	0.962	0.973	0.974
No pretrain	0.882* (p<0.001)	0.955 (p=0.051)	0.957* (p<0.001)	0.954* (p<0.001)
No self-supervising loss	0.948	0.953* (p=0.012)	0.934* (p<0.001)	0.931* (p<0.001)
No self-training loss	0.948	0.954 (p=0.085)	0.958* (p<0.001)	0.960* (p=0.001)
No correction step	0.948	0.956 (p=0.061)	0.962* (p=0.003)	0.962* (p<0.001)

Supplementary Fig. S3. Ablation studies on the components of the proposed method. (a) (b) When ablating each component in the proposed framework, the performance dropped significantly, suggesting the indispensability of the components to attain the optimal performance. Data are presented with calculated area under the receiver operating characteristics curves (AUCs) in the study population (center lines) $\pm$ 95% confidence intervals calculated with the DeLong's method (shaded areas). The AUCs of the proposed method was compared with the other methods at each time point T with the DeLong test to evaluate statistical significance, except for the $T = initial$ where all methods other than no pretrain start from the same baseline. * denotes statistically significant ($p < 0.050$) superiority of the proposed framework. All statistical tests were two-sided.

Supplementary References

1. Chen, T., Kornblith, S., Norouzi, M. & Hinton, G. A simple framework for contrastive learning of visual representations. In *International conference on machine learning*, 1597–1607 (PMLR, 2020).

2. He, K. *et al.* Masked autoencoders are scalable vision learners. *arXiv preprint arXiv:2111.06377* (2021).

3. Caron, M. *et al.* Emerging properties in self-supervised vision transformers. *arXiv preprint arXiv:2104.14294* (2021).

4. Li, X. *et al.* Learning to self-train for semi-supervised few-shot classification. *Advances in Neural Information Processing Systems* **32** (2019).

5. Yalniz, I. Z., Jégou, H., Chen, K., Paluri, M. & Mahajan, D. Billion-scale semi-supervised learning for image classification. *arXiv preprint arXiv:1905.00546* (2019).

6. Xie, Q., Luong, M.-T., Hovy, E. & Le, Q. V. Self-training with noisy student improves imagenet classification. In *Proceedings of the IEEE/CVF Conference on Computer Vision and Pattern Recognition*, 10687–10698 (2020).

7. He, J., Gu, J., Shen, J. & Ranzato, M. Revisiting self-training for neural sequence generation. *arXiv preprint arXiv:1909.13788* (2019).

8. Hinton, G., Vinyals, O. & Dean, J. Distilling the knowledge in a neural network. *arXiv preprint arXiv:1503.02531* (2015).

9. Irvin, J. *et al.* Chexpert: A large chest radiograph dataset with uncertainty labels and expert comparison. In *Proceedings of the AAAI conference on artificial intelligence*, vol. 33, 590–597 (2019).

10. Grill, J.-B. *et al.* Bootstrap your own latent: A new approach to self-supervised learning. *arXiv preprint arXiv:2006.07733* (2020).

11. Caron, M. *et al.* Unsupervised learning of visual features by contrasting cluster assignments. *arXiv preprint arXiv:2006.09882* (2020).
