# Supplementary material for: Self-evolving vision transformer for chest X-ray diagnosis through knowledge distillation
Source: Nat Commun. 2022 Jul 4;13:3848. doi: 10.1038/s41467-022-31514-x (PMC9252561; doi:10.1038/s41467-022-31514-x)
Supplement: Supplementary file 2 — Reporting Summary [file 41467_2022_31514_MOESM2_ESM.pdf]

nature portfolio

Corresponding author(s): Jong Chul Ye

Last updated by author(s): Jun 10, 2022

Reporting Summary

Nature Portfolio wishes to improve the reproducibility of the work that we publish. This form provides structure for consistency and transparency in reporting. For further information on Nature Portfolio policies, see our Editorial Policies and the Editorial Policy Checklist.

Statistics

For all statistical analyses, confirm that the following items are present in the figure legend, table legend, main text, or Methods section.

n/a	Confirmed	
☐	☒	The exact sample size (n) for each experimental group/condition, given as a discrete number and unit of measurement
☒	☐	A statement on whether measurements were taken from distinct samples or whether the same sample was measured repeatedly
☐	☒	The statistical test(s) used AND whether they are one- or two-sided<br>*Only common tests should be described solely by name; describe more complex techniques in the Methods section.*
☐	☒	A description of all covariates tested
☒	☐	A description of any assumptions or corrections, such as tests of normality and adjustment for multiple comparisons
☐	☒	A full description of the statistical parameters including central tendency (e.g. means) or other basic estimates (e.g. regression coefficient) AND variation (e.g. standard deviation) or associated estimates of uncertainty (e.g. confidence intervals)
☐	☒	For null hypothesis testing, the test statistic (e.g. F, t, r) with confidence intervals, effect sizes, degrees of freedom and P value noted<br>*Give P values as exact values whenever suitable.*
☒	☐	For Bayesian analysis, information on the choice of priors and Markov chain Monte Carlo settings
☒	☐	For hierarchical and complex designs, identification of the appropriate level for tests and full reporting of outcomes
☒	☐	Estimates of effect sizes (e.g. Cohen's d, Pearson's r), indicating how they were calculated

Our web collection on statistics for biologists contains articles on many of the points above.

Software and code

Policy information about availability of computer code

Data collection	For open-sourced data, no software was used for data collection. For institutional data, in-house programs were used for de-identification of image data. Python (3.8.5) was used for image pre-processing.
Data analysis	We used Python (3.8.5), Pytorch (1.8.0), Numpy (1.22.2), Pillow (9.0.1), Opencv-python (4.5.5.62), timm (0.5.4), scikit-learn (1.0.2) for the analysis of data with CUDA (11.1) on NVIDIA Quadro 6000, GeForce RTX 3090, and RTX 2080 Ti. Regarding the details of code and algorithm, please refer to the Method section. The source code of model development and validation is deposited at Github: https://github.com/sangjoon-park/AI-Can-Self-Evolve.

For manuscripts utilizing custom algorithms or software that are central to the research but not yet described in published literature, software must be made available to editors and reviewers. We strongly encourage code deposition in a community repository (e.g. GitHub). See the Nature Portfolio guidelines for submitting code & software for further information.

Data

Policy information about availability of data

All manuscripts must include a data availability statement. This statement should provide the following information, where applicable:

- Accession codes, unique identifiers, or web links for publicly available datasets
- A description of any restrictions on data availability
- For clinical datasets or third party data, please ensure that the statement adheres to our policy

Part of CXRs are compiled from publicly available open-source data repositories. The CheXpert repository is available at https://stanfordmlgroup.github.io/competitions/chexpert/, The BIMCV repository is available at https://github.com/BIMCV-CSUSP/BIMCV-COVID-19. The India tuberculosis repository can be found at https://www.kaggle.com/raddar/chest-xrays-tuberculosis-from-india. Montgomery and Shenzen data can be requested via the contact on the follwing webpage

https://openi.nlm.nih.gov/. Belarus tuberculosis repository is available at https://github.com/frapa/tbcnn/tree/master/belarus. The PADChest repository is available at https://github.com/auriml/Rx-thorax-automatic-captioning. The TBX 11K repository can be accessed at https://www.kaggle.com/usmanshams/tbx-11. NIH normal data can be found at https://cloud.google.com/healthcare-api/docs/resources/public-datasets/nih-chest and NIH tuberculosis data can be available at https://tbportals.niaid.nih.gov/downloaddata after getting permission from TB portal. SIIM-ACR Pneumohtorax Segmentation dataset is available at the following repository https://www.kaggle.com/c/siim-acr-pneumothorax-segmentation. Brixia COVID-19 data repository can be found at https://brixia.github.io/. Other part of institutional data ,which were used with institutional permission through IRB approval for this study, are not publicly available due to the patient privacy obligation. Interested users can request the access to these data for research, by contacting the corresponding author J.C.Y (jong.ye@kaist.ac.kr). Any access to de-identified institutional data requires IRB approval at the requesting institution along with the signed agreement on data transfer and usage. Replies to initial request will be made within 10 working days and follow-up based on the answers will be made within institutional review cycles. Use of data is limited to research purposes and redistribution of data is not allowed. Source data are provided with this paper.

Field-specific reporting

Please select the one below that is the best fit for your research. If you are not sure, read the appropriate sections before making your selection.

☒ Life sciences ☐ Behavioural & social sciences ☐ Ecological, evolutionary & environmental sciences

For a reference copy of the document with all sections, see nature.com/documents/nr-reporting-summary-flat.pdf

Life sciences study design

All studies must disclose on these points even when the disclosure is negative.

Sample size	No formal sample size calculation was performed due to the nature of this proof-of-concept study.
Data exclusions	For the institutional data, lateral view images were excluded according to the pre-established criteria, as it is not appropriate to diagnose tuberculosis, pneumothorax and COVID-19 in this view.
Replication	We performed the evaluation in three external datasets collected from totally different environment to examine reproducibility of our finding in various settings. In addition, we tested our model in two different chest X-ray diagnosis tasks including pneumothorax and COVID-19 diagnosis to further verify the reproducibility of our finding in different tasks. We also repeated our experiment with different random division to labeled and unlabeled subsets with different random seeds. All these attempts at replication were successful.
Randomization	The data for model development are randomly divided into one labeled (10%) and three unlabeled datasets (90%).
Blinding	When dividing the data into labeled and unlabeled subsets, the annotations for unlabeled subset are blinded. Other blinding was not applicable as the purpose of this study is to prove that the AI model performance can gradually improved with increasing number of cases accumulated every year without any annotation.

Reporting for specific materials, systems and methods

We require information from authors about some types of materials, experimental systems and methods used in many studies. Here, indicate whether each material, system or method listed is relevant to your study. If you are not sure if a list item applies to your research, read the appropriate section before selecting a response.

Materials & experimental systems

n/a	Involved in the study	
☒	☐	Antibodies
☒	☐	Eukaryotic cell lines
☒	☐	Palaeontology and archaeology
☒	☐	Animals and other organisms
☐	☒	Human research participants
☒	☐	Clinical data
☒	☐	Dual use research of concern

Methods

n/a	Involved in the study	
☒	☐	ChIP-seq
☒	☐	Flow cytometry
☒	☐	MRI-based neuroimaging

Human research participants

Policy information about studies involving human research participants

Population characteristics	The participants from four hospitals are consist of 46.9% males, 42.2% females with median age of 51 years old (range, 6 - 95 years old). Gender and age information were not available in 10.9% and 2.1% of the subjects. Among the chest X-ray obtained from these participants, the images with normal, tuberculosis, pneumothorax, COVID-19 infection, nodule, pleural effusion, interstitial lung disease, bacterial infection were 68.4%, 5.6%, 0.8%, 4.5%, 6.1%, 5.3%, 5.9%, 3.4%, respectively.
Recruitment	Data from the patients who underwent chest X-ray in four hospitals from August 2007 to November 2020 were reviewed by the board certified radiologists. When there is discrepancy on reading between the radiologists, the consensus was obtained.

	The chest X-ray images suggesting normal, tuberculosis, pneumothorax, COVID-19 infection, and other common abnormalities were selected. This may bring in a potential selection bias as the ratio of abnormal over normal cases are higher than real world prevalence, which may increase positive predictive value and decrease negative predictive value in experimental setting compared with real clinical setting.
Ethics oversight	This study was approved by the institutional review boards (IRBs) of Asan Medical Center, Chungnam National University Hospital, Yeungnam University Hospital, Kyungpook National University Hospital, Seoul National University Hospital, and the requirement for informed consent was waived due to the retrospective study design.

Note that full information on the approval of the study protocol must also be provided in the manuscript.
